# Photodynamic Therapy in Combination with Doxorubicin Is Superior to Monotherapy for the Treatment of Lung Cancer

**DOI:** 10.3390/biomedicines10040857

**Published:** 2022-04-06

**Authors:** Joseph C. Cacaccio, Farukh A. Durrani, Joseph R. Missert, Ravindra K. Pandey

**Affiliations:** 1 Photodynamic Therapy Center, Cell Stress Biology, Roswell Park Comprehensive Cancer Center, Buffalo, NY 14263, USA; joseph.cacaccio@roswellpark.org (J.C.C.); farukh.durrani@roswellpark.org (F.A.D.); 2 Photolitec, LLC, 73 High Street, Buffalo, NY 14223, USA; jmissert@photolitec.org

**Keywords:** photosensitizers, photodynamic therapy, toxicokinetics, chemotherapy, combination therapy

## Abstract

We have previously shown that a radioactive (^123^I)-analog of methyl 3-(1′-(iodobexyloxy) ethyl-3-devinylpyropheophorbide-a (PET-ONCO), derived from chlorophyll-a can be used for positron emission tomography (PET) imaging of a variety of tumors, including those where ^18^F-FDG shows limitations. In this study, the photodynamic therapy (PDT) efficacy of the corresponding non-radioactive photosensitizer (PS) was investigated in a variety of tumor types (NSCLC, SCC, adenocarcinoma) derived from lung cancer patients in mice tumor models. The in vitro and in vivo efficacy was also investigated in combination with doxorubicin, and a significantly enhanced long-term tumor response was observed. The toxicity and toxicokinetic profile of the iodinated PS was also evaluated in male and female Sprague-Dawley rats and Beagle dog at variable doses (single intravenous injections) to assess reversibility or latency of any effects over a 28-day dose free period. The no-observed-adverse-effect (NOAEL) of the PS was considered to be 6.5 mg/kg for male and female rats, and for dogs, 3.45 mg/kg, the highest dose levels evaluated, respectively. The corresponding plasma C_max_ and AYC_last_ for male and female rats were 214,000 and 229,000 ng/mL and 3,680,000 and 3,810,000 h * ng/mL, respectively. For male and female dogs, the corresponding plasma C_max_ and AYC_last_ were 76,000 and 92,400 ng/mL and 976,000 and 1,200,000 h * ng/mL, respectively.

## 1. Introduction

Among a variety of cancer types, lung cancer is considered to be the leading cause of death related and the most commonly diagnosed form of such disease [1,2]. Lung cancer is divided into two broad histologic classes, which grow and spread differently: small-cell lung carcinomas (SCLCs) and non-small cell lung carcinomas (NSCLCs) [3]. Treatment options for lung cancer include surgery, chemotherapy and radiation therapy [4,5,6,7]. However, in many cases, cancer cells develop drug resistance and become nonresponsive to chemotherapy [8], thus necessitating the exploration of alternative and/or complementary treatment modalities. Photodynamic Therapy (PDT) has emerged as an effective treatment modality for various malignant neoplasia and tumors [9,10]. In PDT, the photochemical interaction of light, photosensitizer (PS) and molecular oxygen produces reactive oxygen species (ROS), mainly singlet oxygen (^1^O_2_), which is responsible for the destruction of tumor [11,12,13,14,15].

A large number of porphyrin-based photosensitizers (PS) has been investigated in-clinic for the treatment of lung cancer by PDT [16,17,18,19,20,21,22], and the initial response has been encouraging. However, some of the first-generation PSs showed limited tumor specificity and prolonged skin phototoxicity. Moreover, PDT being a localized treatment was not curative for those patients with metastasis. In most of the second-generation agents, especially with HPPH [3-(1′-hexyloxy) ethyl-3-devinylpyropheophorbide-a] [23], derived from chlorophyll-a, the long-term skin phototoxicity problem has been resolved [24], but it is potentially curative only for localized cancers. Therefore, efforts are currently underway to investigate the utility of PDT in combination with other treatment modalities, e.g., chemotherapy, immunotherapy, etc. [25,26]. The initial clinical results are promising but the treatment parameters need to be optimized in a large patient population.

For the past several years, one of the objectives of our laboratory has been to develop multi-functional agents for cancer-imaging (PET, MRI or fluorescence or of these combination) [27] and treatment of cancer by PDT, using a “See and Treat” approach. In one of our attempts, we have been able to develop an iodinated PS (methyl-3(1′-m-iodobenzyloxy) ethyl-3-devinyl pyropheophorbide-a), which in its radioactive form (^124^I-) can be used to image a variety of tumors by PET imaging [28], and as a non-radioactive analog for NIR fluorescence-imaging and treatment of cancer by PDT. Thus, a single agent (in combination of radioactive + corresponding non-radioactive forms) can be used for imaging (PET, fluorescence) and therapy of cancer [28]. This product provides a unique opportunity to determine the stage of cancer (localized or metastasized) by PET imaging of the cancer patient with ^124^I-labeled agent and select the treatment plan accordingly: either PDT alone (if cancer is localized) or PDT + chemotherapy (if the cancer is metastasized). Therefore, we initially investigated the PET imaging ability of the ^124^I-labeled agent of this compound (PET-ONCO) in a variety of tumor types, including lung tumors, and excellent results were obtained [28]. This report presents (a) the utility of a corresponding non-labeled iodinated PS **1** for treating lung cancer with and without chemotherapy (doxorubicin) in a variety of lung tumors xenografts derived from lung cancer patients and (b) the toxicity and toxicokinetic profiles of the PS formulated in Pluronic F-127 at variable doses in male and female rats and dogs. We and others have previously shown the improved PDT efficacy of certain tetrapyrrolic photosensitizers in a Pluronic-based formulation either by encapsulation or by conjugating the PS with Pluronic F-127 with and without the combination of co-delivery of doxorubicin for overcoming drug resistance in cancer [29,30,31].

## 2. Results and Discussion

**Figure 1:**
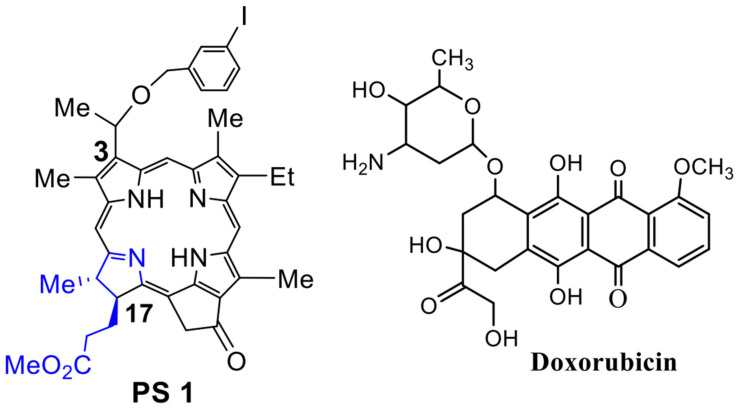
Doxorubicin, a chemotherapy agent [32] routinely used for the treatment of lung cancer patients, was purchased from Sigma Aldrich, USA.

**Chemistry:** The PS **1** [(methyl-3-(1′-*meta*-iodo-benzyloxy) ethyl-3-devinylpyropheophorbide-a] was synthesized from Chlorophyll-a by following the methodology established in our laboratory [28]. See Figure 1.

***In vitro Studies:***

**Figure 2:**
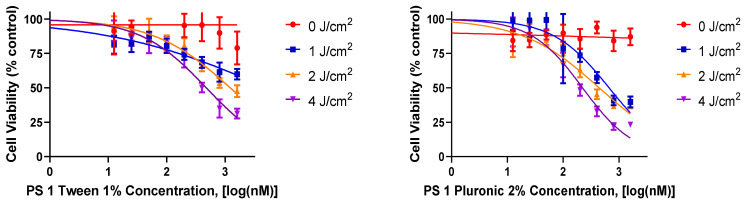
A comparative in vitro PDT efficacy of PS **1** formulated either in 1% Tween^®^-80 (left graph) or 2% Pluronic^®^ F-127 (right graph) in lung cancer cell line 14541 derived from a lung cancer patient. The cells were incubated with PS **1** for 24 h, and then exposed to light (665 nm, 1–4 J/cm^2^) 24 h. The PDT efficacy was determined by MTT assay, and the results were analyzed using GraphPad Prism 7 software.

*(a)*  *In vitrocell uptake and PDT efficacy of PS1 in Tween80 vs Pluronic F 127 formulations:* For *in vitro* studies, PDX 14541 cell line (a squamous cell carcinoma, SCC), derived from a lung cancer patient tumor was initially used to investigate the PDT efficacy of iodinated photosensitizer (PS) **1**. The PS was formulated in two different formulations (1% Tween 80/5% dextrose and 2% Pluronic F-127 in PBS) to determine the impact of delivery vehicle in PDT efficacy at various light and drug doses. Among the parameters used, PS **1** formulated in 2% Pluronic F-127 showed significantly higher efficacy when compared to the 1% Tween 80 formulation (Figure 2). At the light dose of 1 J/cm^2^ (665 nm), the IC_50_ values of PS **1** in Pluronic and Tween80 formulations were 662.5 nM and 5196 nM, respectively. Finally, neither formulation showed any dark toxicity with drug alone and no light treatment.

**Figure 3:**
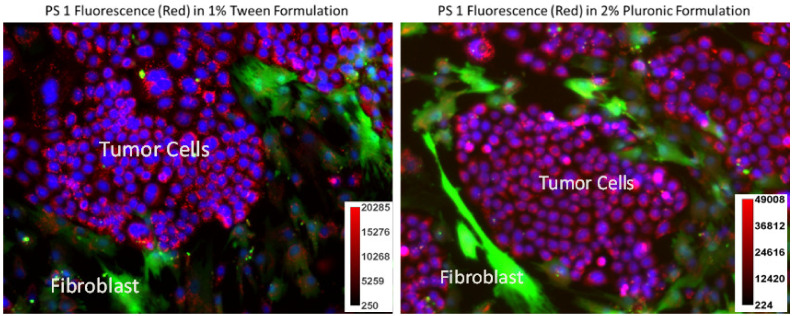
A co-culture system containing PDX 14541 tumor cells and normal lung-fibroblast cells transfected with GFP. PS **1** uptake (red) was observed in both Tween^®^-80 and Pluronic^®^ F-127 formulations within the tumor cells. GFP-transfected fibroblast cells are shown in green. While both tumor cells and fibroblast demonstrated PS fluorescence, the PS concentration amount (fluorescence intensity) observed in normal fibroblast cells was significantly lower than in tumor cells. Compared to Tween 80 formulation, the PS **1** in Pluronic F-127 formulation showed more evenly PS distribution. Hoechst 33342 was used to stain the nucleus of the cells (blue). The PS **1** did not show any localization in cell nucleus. The fluorescence intensity of the PS **1** in Tween and Pluronic formulations was measured by ImageJ software (see scale bars).

*(b)*  *Impact of PS **1** formulated in Tween and Pluronic in PDX 14541 cells and fibroblast co-culture:* Photosensitizers which specifically accumulate in tumor cells over normal cells is vital in minimizing adverse effects. To demonstrate PS **1** tumor specificity over normal lung cells, a co-culture system was prepared using PDX 14541 cells and normal lung fibroblast. Additionally, the normal lung fibroblast cells were transfected with GFP to distinguish the two cell types visually. In this system, PS **1** in both formulations (Tween^®^-80 and Pluronic^®^ F-127) showed higher uptake in tumor cells over the normal cells. However, the Pluronic formulation had a better distribution across the tumor cells mass. In the Tween formulation, the PS concentration along the periphery of the tumor cell mass was higher compared to the center of the mass determined by its fluorescence intensity (Figure 3).

**Figure 4:**
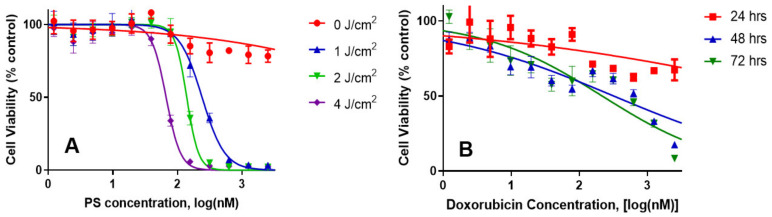
The in vitro cytotoxic effect of PS **1**-PDT at variable concentrations/light doses and doxorubicin efficacy at variable concentrations after incubating for 48 h were independently determined in A549 cell lines. For the PDT experiment, the cells were exposed to light at 24 h. Cells were then rested for 48 h and tested for cytotoxic effects via MTT assay. The cells were incubated with doxorubicin for 48 h and then tested for cytotoxicity by MTT assay.

*(c)*  *Comparative independent**in vitro efficacy of PS 1-PDT and doxorubicin therapy:* PDT is an efficient modality in destroying localized tumors but has limitations in treating metastasis, where the delivery of the light could be problematic. To demonstrate the advantages of PDT in combination with doxorubicin, and its synergetic impact to treat lung cancer, the Bliss independence model of synergy was investigated in A549 lung cancer cells. The tumor cells were incubated with PS **1** at variable concentration for 24 h, washed with fresh media and exposed to variable light doses (1–4 J/cm^2^), and the PDT efficacy was determined by MTT assay [33]. For determining the efficacy of doxorubicin, the A549 cells were treated with doxorubicin at variable concentration, incubated for 24, 48 or 72 h. The effective dose was determined via the MTT assay (Figure 4). The IC_50_ values of the PS (conc. 300 nM), light dose (665 nm, 1 J/Cm^2^) at 24 h post-incubation of the PS) and doxorubicin (625 nm, cells incubated for 48 h) were used to select the concentration of the PS and doxorubicin for determining the best treatment parameters.

**Figure 5:**
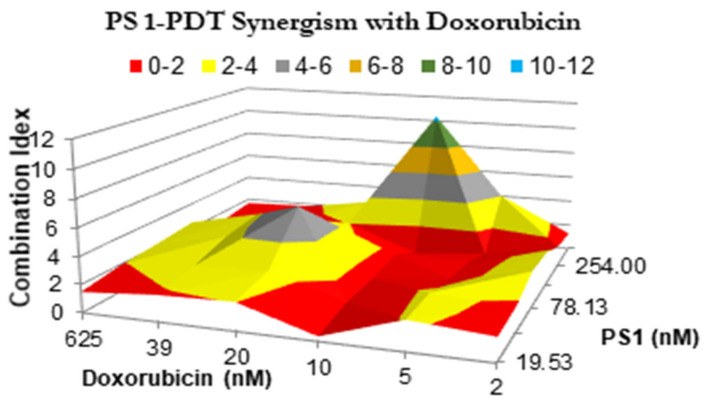
The synergism of PS **1** and doxorubicin were tested at varying drug doses. A549 cells were incubated with PS **1**. Cells were exposed to light (fluence: 1.0 J/cm^2^, fluence rate: 75 mW/cm^2^) applied at 24 h post-incubation. Doxorubicin was then added at various concentrations, and cells were incubated for 48 h, then assessed for cytotoxic effects. The combination indices were calculated for each point using the formula CI = [E_PS1_ + E_dox_ − E_PS1_E_dox_]/E_PS1*dox_. Therefore, the combination index plotted above represents the ratio between the hypothetical efficacy and the observed efficacy of the combination. Peaks indicate antagonism, values near 1.0 indicate an additive effect, and valleys indicate synergism.

*(d)*  *PS **1**-PDT in combination with doxorubicin therapy shows a synergetic effect. * The impact of PS **1**-PDT in combination with doxorubicin was studied and analyzed by following the Bliss method. [34]. The Bliss independence model was generated by comparing the cell viability data from individual treatment, with those obtained by combining both the modalities in various PS and doxorubicin doses. A topological map was generated by graphing 36 different peaks of antagonism followed by valleys of synergy, indicated in red (Figure 5). A combination index that is less than 1 indicates synergy, while if the value is greater than 1, the combination was antagonistic. The in vitro model suggests that for best efficacy, doxorubicin and PS should be used in a molar ratio of 2:1. In a combination therapy experiment, the cell viability was reduced to 50% at 20 nM conc. of PS **1** and 10 nM conc. of doxorubicin, whereas PS **1** alone at 20 nM yielded 90% viability and doxorubicin alone at 10 nM showed 80% cell viability.

**Figure 6:**
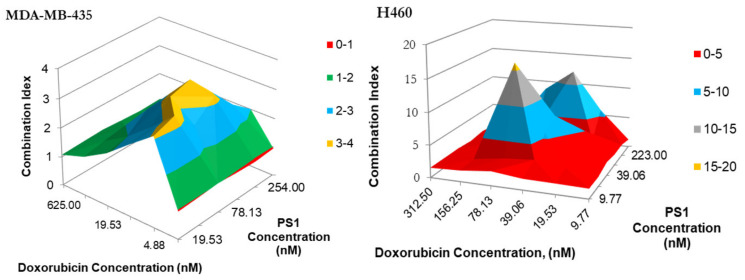
The synergism of PS **1** and doxorubicin tested MDA-MB-435 in H460 and at varying drug doses. Cells were plated and PS **1** was added. Cells were exposed to light (fluence: 1.0 J/cm^2^, fluence rate: 75 mW/cm^2^) at 24 h post-incubation. Doxorubicin at various doses was then added, and cells were incubated for 48 h, then assessed for cytotoxic effects. The combination indices were calculated for each point using the formula CI = [E_PS1_ + E_Dox_ − E_PS1_E_Dox_]/E_PS1+Dox_. Peaks indicate antagonism, values near 1.0 indicate an additive effect, and valleys < 1.0 indicate synergism. Areas indicated in red denote synergetic concentrations and valleys < 1.0 indicate synergism. Areas indicated in blue and red denote synergetic concentrations.

**Figure 7:**
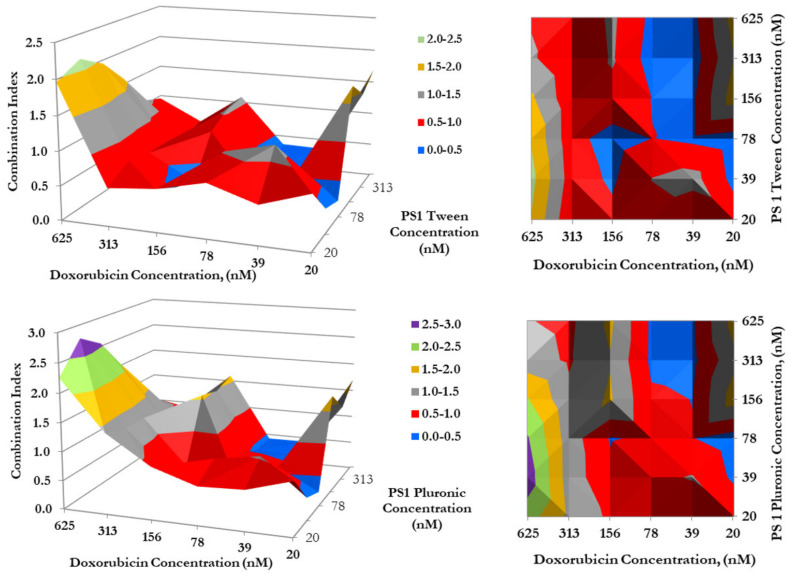
The synergism of doxorubicin in combination with PS **1** in either Tween or Pluronic formulation. Cells were plated and PS **1** was added. Cells were exposed to light (Fluence: 1.0 J/cm^2^, Fluence rate: 75 mW/cm^2^) at 24 h post-incubation. Doxorubicin was then added at variable concentrations, and cells were incubated for 48 h, then assessed for cytotoxic effects. The combination indices (C.I.) were calculated for each point using the formula C.I. = [E_PS1_ + E_Dox_ − E_PS1_E_Dox_]/E_PS1*Dox_. Peaks indicate antagonism, values near 1.0 indicate an additive effect, and valleys <1.0 indicate synergism. Areas indicated in red and blue denote synergetic concentrations, while grey indicates an additive effect.

*(e)*  *Synergetic Impact of PS **1**-PDT and doxorubicin in various cell lines: * Before initiating in vivo studies, additional in vitro experiments were preformed to investigate if synergetic trends remained consistent in two additional cell lines: H460 (non-small cell lung cancer) and MDA-MB-43 (breast cancer). H460 reacted similarly to A549, except the regions of antagonism were expanded. This includes the 2:1 combination yielding the highest degree of synergy. Meanwhile, MDA-MB-435 demonstrated almost no areas of synergy. Instead, most of the combinations yielded additive or antagonistic effects (Figure 6 and Figure 7).

*(f)*  *Impact of formulation (Tween* vs. *Pluronic formulation of PS) in combination with doxorubicin in combination therapy:* To determine if the photosensitizer delivery vehicle(s) had any influence in the mode of action of PDT in combination with doxorubicin therapy, a synergetic study was conducted by using both the formulations of PS **1**. The PS **1** dissolved in Tween80/5% Dextrose/ D5W yielded similar synergy with doxorubicin, as shown previously in Pluronic formulation (Figure 5), where the highest synergetic effect was observed when PS **1** and doxorubicin concentrations were in a ratio of 2:1.

***In vitro Studies:***

*(a)*  *PS 1 shows high tumor-specificity and stability in Pluronic (2%) formulation* Similar to most of the porphyrin-based compounds the iodinated PS **1** also showed limited solubility in water. Therefore, it was formulated in two FDA approved formulations: (i) Tween 80/dextrose in water and (ii) Pluronic F-127/PBS at various concentrations, and the stability/concentration of PS in formulation solution was determined at 4 °C and −20 °C. In both formulations (1%Tween 80/5% Dextrose and 2% Pluronic/PBS), the photosensitizer could be dissolved in a high concentration, and was stable, with no loss of PS concentration at least for 24 months at −20 °C. PS **1** can also be formulated at lower concentrations of Pluronic (0.5%, 1.0%), but the long-term stability was low with a significant release of the PS. The concentration/stability/purity of PS **1** in both formulations was confirmed by spin filtration of the formulation, and then analyzing the filtrate(s) and retentate for the concentration of the PS by spectrophotometric and HPLC analyses.

**Figure 8:**
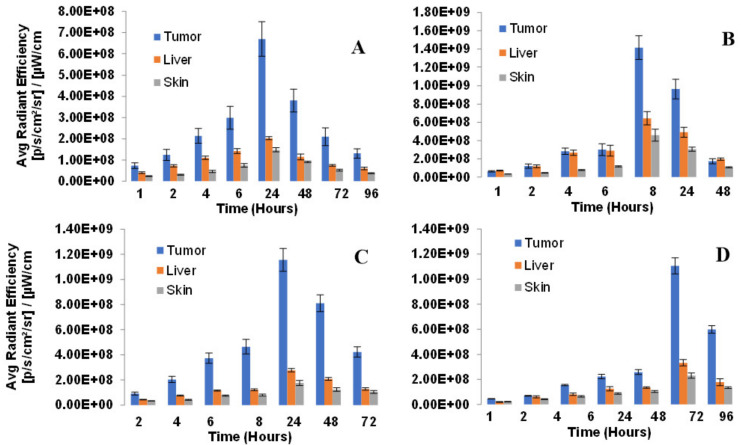
Biodistribution of PS in lung PDX models: (**A**) NSCLC148070, (**B**) NSCLC 0229047, (**C**) SCC 14541 and (**D**) Lung Adenocarcinoma 15021. SCID mice (3 mice/group) were implanted with lung xenografts on the right flank. Tumors were grown until reaching approximately 5 mm diameter. PS **1** (formulated in 2% Pluronic F-127, at a dose of 0.47 µmol/kg was administered retro-orbitally (alternate for tail vein injection). Tumor, liver and skin uptake was measured by an IVIS Spectrum using Living Image acquisition and analysis software at 2, 4, 6, 8, 24, 48 and 72 h. Excitation and emission filters were 640–660 nm and >720 nm respectively. In all tumor types, the maximum uptake was observed at 24 h post-injection of the PS.

To establish the treatment parameters of PDT (especially the optimal time for light irradiation to tumors), whole body fluorescence imaging of the desired PS over variable timepoints was performed in four PDX models (NSCLC 148070, NSCLC 0229042, SCC 14541 and lung Adenocarcinoma 15021). The mice (SCID, 3 mice/group) were injected with the PS **1** (0.47 mmol/kg) formulated in 2% Pluronic F-127/PBS and the whole-body fluorescence imaging was performed via epi-illumination on an IVIS-in vivo system. Image analysis was carried out with Living Image Acquisition and Analysis Software. The fluorescence was measured using an excitation wavelength at 640 nm and emission at 680 nm as the instrument was most sensitive to detect fluorescence of PS using this filter set. Images were analyzed for average radiant efficiency over three regions of interest (ROI) covering the tumor, liver and skin, and results were expressed as the mean average radiant efficiency +/− standard deviation. The highest fluorescence was observed at 24 h post-injection in all tumor models. Interestingly, the greatest difference in PS uptake in tumor vs. liver and skin determined by fluorescence imaging was also seen at the same time point (Figure 8).

*(b)*  *Determination of PDT Efficacy of PS **1** in PDX Models * We have previously investigated the in vivo PDT efficacy of **PS 1** in mice bearing FaDu, Colon26, UMUC3 and U87 tumors, and the most effective drug dose was determined to be 1.0 mmol/kg, and light dose: 135 J/cm^2^, 75 mW/cm^2^. Therefore, we used the same treatment parameters for evaluating its efficacy in a variety of lung PDX models (NSCLC148070, NSCLC 15021, SCLC14541, SCLC 0229047).
*(c)*  Imaging of PDX tumors Small Cell Lung Carcinoma (SCLC) and Non-Small Cell Lung Carcinoma (NSCLC): SCLC (A) 14541 (B) 0229047 and NSCLC (C) 15021 (D) 148070 using PS1 at 0.47 µmol PS **1** in 2% Pluronic^®^ F-127: Similar to most of the pyropheophorbides, PS **1** is also a highly fluorescent molecule with long wavelength absorption at 665 nm and emission at 670 nm/720 nm.

The success of PDT depends on the PS uptake and retention in tumor(s), availability of oxygen and exposure with an appropriate wavelength of light. For the present study, the female SCID mice were implanted with xenografts on the right flank. The tumors were grown until reaching approximately 5 mm diameter. The PS was then injected intravenously at a dose of 1 µmol/kg and whole-body fluorescence imaging via epi-illumination was performed by an IVIS Spectrum system. Image analysis was carried out with Living Image acquisition and analysis software. The image obtained using Ex: 640–660 nm, Em: >680 nm was used as the instrument was most sensitive to detect fluorescence of PS 1 using this filter set. Images were analyzed for average radiant efficiency over three regions of interest (ROIs) covering the tumor, liver and skin (shaved), and results were expressed as the mean average radiant efficiency ± standard deviation. The time for highest uptake of the PS was determined by fluorescence at variable time points after injecting PS **1**.

**Figure 9:**
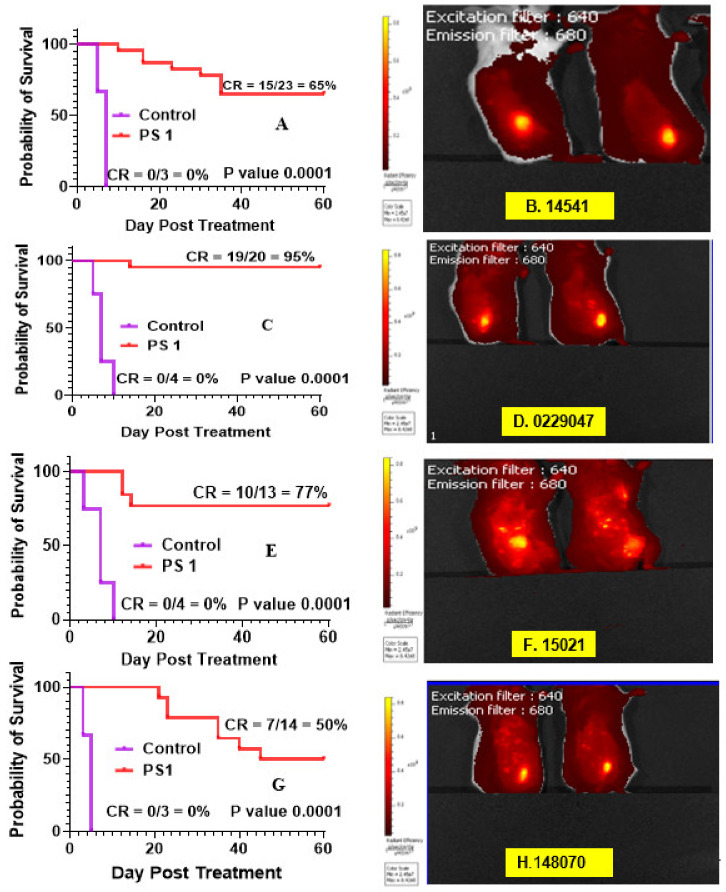
Fluorescence imaging and PDT efficacy of SCID mice bearing PDX lung tumors: (**A**) PDT efficacy of mice bearing SCLC 14541 tumors and (**B**) tumor images; (**C**) PDT efficacy of mice bearing SCLC 2229047 tumors and (**D**) tumor images**;** (**E**) PDT efficacy of mice bearing NSCLC 15021 tumors and (**F**) tumor images; (**G**) PDT efficacy of mice bearing NSCLC 148070 tumor and (**H**) tumor images. For determining long-term efficacy, mice were injected (i.v.) with PS **1** (1 mmol/kg), and at 24 h post-injection, the tumors were exposed with light (665 nm, 135 J/cm^2^, 75 mW/cm^2^), and tumor growth was monitored daily for 60 days.

*(d)*  *PDT efficacy of PS **1** in treating SCLC PDX tumors: 14541 and 0229047: * PDT treatment was performed and replicated using 1 µmol/kg PS **1** in 2% Pluronic^®^ F-127 in female SCID mice bearing SCLC tumors irradiated with light at 665 nm at a dose of 135 J/cm^2^ and fluence rate of 75 mW/cm^2^ (Figure 9). Mice were followed for 60 days post-PDT treatment, and palpable tumors were monitored via caliper measurement. Tumor volume was calculated as length x width x ½ width. The cure rates (CR) in both SCLC tumors at 60 days were 15/23 = 65% and 19/20 = 95%, respectively, with a significant *p* value of 0.0001 in both PDX types.

**Figure 10:**
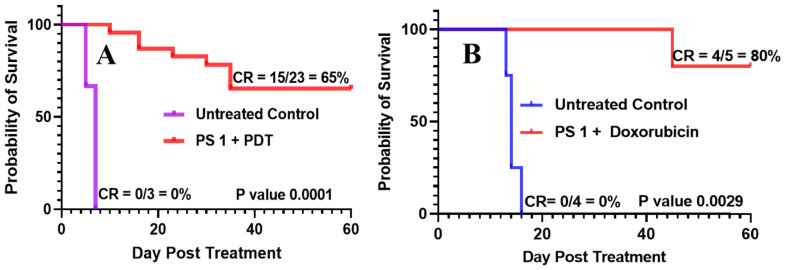
(**A**) SCID mice were injected intravenously with PS **1** at a dose of 1.0 μmol/kg; the tumors were irradiated with light (665 nm, light dose: 135 J/cm^2^, 75 mW/cm^2^) at 24 h post-injection. (**B**) In a second set of experiments**,** five SCID mice bearing SCLC 14541 tumors after PDT treatment as discussed above were injected (i.v.) with doxorubicin (2.5 mg/kg × 1 dose), and tumor regrowth of mice in both sets of experiments was monitored daily for 60 days. If there is any tumor regrowth, the mice were euthanized on that day. On day 60, mice with no tumor regrowth (cure) were also euthanized following an approved protocol procedure.

*(e)*  *PDT efficacy of PS **1** in treating of NSCLC PDX tumors: 15,021 and 48,070: * PS **1-**PDT was also evaluated in SCID mice bearing SCLC tumors (PDX 15,021 and 48,070) at a dose of 1.0 µmol/kg in 2% Pluronic^®^ F-127. At 24 h post-injection of the PS, the tumors were irradiated with light at 665 nm at a light dose of 135 J/cm^2^ and fluence rate of 75 mW/cm^2^. The tumor regrowth in each mouse was followed for 60 days post-PDT treatment, and palpable tumors were monitored via caliper measurement. Tumor volume was calculated as length × width × ½ width. The cure rates (CR) in both NSCLC tumors at day 60 were 10/13 = 77% and 7/14 = 50%, respectively, with a significant *p* value of 0.0001 in both PDX types (Figure 9).
*(f)*  *PS **1**-PDT in combination with doxorubicin enhances long-term tumor cure: * To investigate the impact of PDT in combination with chemotherapy, PDT treatment was performed first using female SCID mice bearing SCLC 14541 tumors (PDX). In brief, PS **1** at a dose of 1 µmol/kg was injected. At 24 h post-injection, the tumors were exposed to light (665 nm, light dose: 135 J/cm^2^, 75 mW/cm^2^) and then the PDT treated mice were injected *(i.v.)* only one time with doxorubicin at a dose of 2.5 mg/kg. Mice were monitored for 60 days post-PDT treatment, and palpable tumors were measured via caliper measurement. Tumor volume was calculated as length × width × ½ width. The cure rates (CR) in SCLC tumors at 60 days were 15/23 = 65% and in combination with doxorubicin were 4/5 = 80%, respectively, with a significant P value calculated by Mantel-Cox software in both treatment types. The tumor response depicted in Figure 10 shows a significant improvement in long-term cure by PDT in combination with doxorubicin therapy.

**Figure 11:**
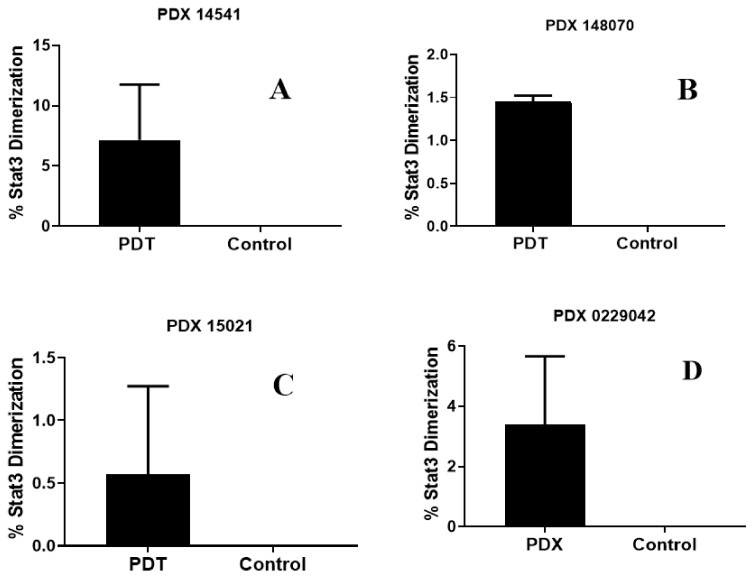
Percentage of STAT3 dimerization measured after in vivo PDT in SCID mice (3 mice/group) bearing tumors on the flank with patient-derived lung cell carcinoma SCC 14541 (**A**), USCLC 148070 (**B**), adenocarcinoma NSCLC 15021 (**C**)**,** and squamous cell carcinoma, SCC 0229047 (**D**). Tumors were grown for approximately 2 weeks until they reached 6–10 mm diameter; the mice were then injected with 1.0 µmol/kg PS **1** in 2% Pluronic^®^ F-127 formulation. In each set of experiments, PDT treatment was given to two mice at 24 h with light at 665 nm at a dose of 135 J/cm^2^ and fluence rate of 75 mW/cm^2^. One mouse in each experiment also received PS **1,** but no light treatment and used as a control.

*(g)*  *STAT3 dimerization as a bio-marker to PDT response:* We have previously shown that the STAT3 dimerization is an efficient biomarker for predicting the outcome of PDT treatment both in vitro and in vivo [35]. Various in vivo experiments conducted in animals, and the clinical PDT using HPPH as a PS have shown that a significant amount of the HPPH was not bleached (destroyed) after the light treatment (PDT), and those patients who showed limited STAT3 dimerization on further light treatment gave improved long-term tumor cure. Thus, STAT3 dimerization could be a valuable biomarker in evaluating the PDT response in cancer patients. Therefore, in this particular study, the percentage of STAT3 dimerization after light treatment was measured in mice bearing various lung cancer tumors, and the preliminary results shown in Figure 11 indicate that all the tumors showed a certain percentage of STAT3 dimerization, but this was not directly proportional to the tumor response (Figure 9). These results are certainly interesting; however, further in vivo studies are needed to confirm a direct correlation between the percentage of STAT3 dimerization and long-term PDT efficacy using a larger group of mice bearing a variety of tumor types with variable vascularity.

*(h)*  *Impact of Pluronic F-127 formulation in photophysical properties of PS **1**: * Similar to most of the tetrapyrrole-based PS, e.g., HPPH^30^, the PS **1** derived from chlorophyll-a, on formulating in Pluronic F-127/PBS solution forms aggregation, which significantly reduces its absorption and fluorescence intensities. However, in the presence of HSA (human serum albumin) or BSA (bovine serum albumin), the PS self-aggregation disaggregates, and exhibit photophysical properties similar to the respective monomers observed in organic solvents (e.g., methanol or tetrahydrofuran).

## 3. PS 1 Toxicity at Variable Doses in Rats and Dogs 

The objectives of this study were to evaluate the toxicity and toxicokinetic profiles following bolus intravenous (IV) administration of PS **1** (formulated in 2% Pluronic F-127/PBS) to male and female Sprague-Dawley rats and Beagle Dogs after a single dose to assess reversibility of latency of any effects over a 28-day free period. These studies were performed under the guidelines set by the United States Food and Drug Administration, in a GMP Facility (Frontage Laboratories, Cleveland, OH, USA).

*(a)*  Rats’ toxicity results: The iodinated PS **1** was administered to male and female rats, by a single iv injection, at target dose levels of 0, 1, 4 or 8 mg/kg (Table 1). The control and high-dose animals were administered 0 or 8 mg/kg of the test article in 2% Pluronic F-127 (*w/v*) in DPBS. The low dose animals were administered 1 mg/kg of the test article in 0.25% luronic F-127 and the mid-dose animals were administered 4 mg/kg of the test article in Pluronic F-127 (2% *w/v* in PBS). The Group 1–4 animals were terminated the day after dose administration (Day 2, ten rats/sex/group) or after a 28-day recovery period (Day 29, ten rats/sex/group). Separate groups 3 rats/sex/group of animals were used for toxicokinetic evaluation (3 rats/sex/group in vehicle control group and 9 rats/sex/group in the drug treatment group).

Analysis of samples collected from the 0.1, 0.4 and 0.8 mg/mL dose formulations demonstrated that these formulations were homogeneous (CV</− 1.4%). The mean concentrations of the 0.1, 0.4 and 0.8 mg/mL homogeneity samples were 111, 83.0 and 81.9% of the target, respectively, which were outside of the accuracy criteria (+/− 10%) of the target concentration. Analysis of backup samples confirmed the initial results. The actual dose levels were 1.11, 3.32 and 6.55 mg/kg, respectively. 

There was no mortality or moribundity and no PS related effects on: clinical observations, body weight, food consumption (recovery animals only), ophthalmology clinical pathology (hematology, coagulation, clinical chemistry and urinalysis), gross pathology, organ weights and histopathology or microscopic findings. 

On day 2, the most notable changes were several-fold elevations in serum cholesterol and triglycerides concentrations for both males and females of Group 1 (vehicle) and Group 4 (high dose). On day 29, both cholesterol and triglycerides values returned to the normal range. These changes were potentially Pluronic F-127 related (a vehicle component). In addition, statistically significant changes were observed in multiple serum chemistry on Day 2. These changes were not considered adverse due to their scattered small magnitude nature. The T_max_ ranged from 0.083 to 1000 h. The elimination half-life ranged from 7.03 to 9.90 h. Increases in C_max_ were approximately dose proportional and increases in ACCl_ast_ were greater than dose proportional. These values were similar in males and females. Single intravenous injections of PS **1** to male and female rats at dose levels of 0, 1.11, 3.32 and 6.55 mg/kg was well tolerated with no adverse test article-related effects. The no-observed-adverse-effect level (NOAEL) for the PS was considered to be 6.55 mg/kg for male and female rats, the highest dose level evaluated (Table 2).

*(b)*  Dogs’ toxicity results**:** Photosensitizer **1** was administered to male and female Beagle dogs, by a single intravenous injection, at dose levels of: 0, 0.5, 2 or 4 mg/kg.

The low-dose animals were administered 0.5 mg/kg or the test article of 0. 25 Pluronic F-127 (*w/v*) in DPBS and the mid-dose animals were administered 2 mg/kg of the test article in 1% Pluronic F-127 (*w/v*) in Dulbecco’s Phosphate Buffered Saline (DPBS). There were 6 dogs/sex in each dose group with 3 dogs/sex terminated the day after the final dose and 3 dogs/sex terminated after a 28-day recovery period. The study design is shown in Table 3.

Analysis of samples collected from 0.1, 0.4 and 0.8 mg/mL formulations demonstrated that these formulations were homogenous (RSD </−). The mean concentrations of the samples were 84.7, 76.0 and 86.3% of the target, respectively, which were outside of the accuracy criteria. The actual dose levels were 0.42, 1.52 and 3.45 mg/kg, respectively.

Similar to the study discussed above on rats, there was no mortality and there were no adverse evaluations in triglycerides levels or moribundity and no PS-related effects on: clinical observations, body weight, food consumption (recovery animals only), ophthalmology clinical pathology (hematology, coagulation, clinical chemistry and urinalysis), gross pathology, organ weights and histopathology or microscopic findings. The triglyceride values returned to within the normal range by Day 29.

The T_max_ ranged from 0.083 to 0.458 h. The elimination half-life was 13.7 h. Increase in C_max_ and AUC_last_ were 76,000 and 92,000 ng/mL and 976,000 and 1,2000,000 hr * ng/mL for males and females, respectively. The no-observed-adverse-effect level (NOAEL) for PS **1** was observed to be 3.45 mg/kg for male and female dogs, the highest dose level evaluated (Table 4).

## 4. Conclusions

The results presented in this article show that the iodinated PS **1** derived from chlorophyll-a is an efficient photosensitizer for the treatment of a variety of l PDX lung cancer tumors. Interestingly, PS**1**-PDT in combination with doxorubicin at a single dose enhanced the long-term cure in SCID mice bearing SCLC 14541 tumors. These results are exciting, and in a future study, the optimization of treatment parameters at variable doses of PS and chemotherapy agents (either doxorubicin or cisplatin) may further improve long-term cure with reduced toxicity. This approach will certainly help to select the best treatment parameter for treating lung cancer patients. The advantages of the iodinated compound are due to its unique ability to image the cancer in radioactive form (^124^I-), and as a non-radioactive analog it can be used for fluorescence guided photodynamic therapy of cancer. A “true” tri-functional (MR, fluorescence imaging and image-guided therapy).

The toxicity and toxicokinetic profiles of PS **1** in 2% Pluronic F-127 formulation was investigated at variable doses in rats and dogs in a GMP facility, following the United States FDA guidelines. Under the doses tested, even at higher than the therapeutic dose, no significant toxicity was observed. 

## 5. Experimental Methods

Chemistry**:** The iodinated PS **1** was derived from chlorophyll-a in a multistep synthesis following our own methodology. [33]. The GMP material for toxicity and toxicokinetic studies in rats and dogs was synthesized in a GMP facility following the guidelines of the United States FDA.

Cell Culture and establishing patient-derived xenograft cell line:  Lung cancer cell lines (A549 and H460) and breast cancer cell line (MDAMB435) were acquired from ATCC. Cells were grown in 75 cm cm^2^ flask with 10% Fetal bovine serum and 5% Penicillin Streptomycin-supplemented media were used to grow the cells under normoxic conditions of 5% CO_2_ at 37 °C.

Patient-derived mouse-carried xenografts (PDX) were isolated and grown as epithelial cell lines. Briefly, to establish a cell line from tumor chunks, the tumors were digested in trypsin and DNase I. The epithelial cells were mechanically separated from the tumor and allowed to grow on collagen coated plates. These PDX cell lines were used to investigate tumor specificity of PET-ONCO compared to normal fibroblast cells as well as investigate PDT efficacy in vitro.

Co-culture system of PDX 14541 cells and normal lung fibroblast:  PDX 14541 tumor cells were plated in a 6-well plate at around 1000 cells per well. After 24 h, normal lung fibroblast cells that had been pre-transfected with GFP (provided by Dr. Heinz Baumann, Molecular & Cellular Biology, Roswell Park Comprehensive Cancer Center) were plated next at about 5000 cells per well. The cells were allowed to grow to confluency and then were dosed with 1 µM PS**1**. Next, 24 h after dosing, the cells were stained with Hoechst 2422 and imaged using a Zeiss fluorescent microscope. 

***Determination of in vivo Imaging/PDT efficacy:***

Fluorescence Imaging: The SCID mice with PDX Lung tumors of 200–250 mm^3^ were injected intravenously (i.v) with photosensitizer PS **1** at dose of 1 µmol/kg in Pluronic formulations. The PS uptake in tumors was determined by fluorescence imaging using a PerkinElmer IVIS Spectrum at variable time points, and maximum uptake was observed at 24 h post-injection. 

PDT Efficacy/Tumor Response: At this timepoint, the tumors were irradiated with light (fluence: 135 J/cm^2^; fluence rate: 75 mW/cm^2^) for 30 min at 665 nm using a Lightwave™ laser diode. Mice were restrained without anesthesia in plexiglass holders designed to expose only the tumor and a 2–4 mm annular margin of skin to light. Two axes (mm) of tumor (L, longest axis; W, shortest axis) were measured with the aid of a Vernier caliper. The tumor assessment and measurements were taken daily, then three times a week for 4 weeks, and twice a week thereafter for a total of 60 days post treatment. Tumor volume (mm^2^) was estimated using a formula: tumor volume = ½ (L × W^2^). The complete tumor regression (CR) was defined as the inability to detect tumor by palpation at the initial site of tumor appearance for more than two-month post-therapy. Partial tumor regression (PR) was defined as ≥50% reduction in initial tumor size. The edema, erythema and scar formation in the treatment field was observed and recorded. Tumor response for each treatment was evaluated for the tumor response. For statistical analysis, the log-rank Mantel-Cox test, a standard analysis method, was used.

## Figures and Tables

**Table 1:** Group assignments and dose levels (rat study).

Dose Group	Number of Animals (M/F)	Test Article	Target		Actual ^a^		Dose Volume (mL/Kg)	Number of Animals For Necropsy (M/F)	
			Dose Level (mg/kg)	Dose Conc. (mg/mL)	Dose Level (mg/kg)	Dose Conc. (mg/kg)			
								Main (Day 2)	Recovery (Day 29)
Toxicity Groups									
1	20/20	PS 1	0	0	0	0	10	10/10	10/10
2	20/20	PS 1	1	0.1	1.11	0.111	10	10/10	10/10
3	20/20	PS 1	4	0.4	3.32	0.332	10	10/10	10/10
4	20/20	PS 1	8	0.8	6.55	0.655	10	10/10	10/10
Toxicokinetic Groups									
5	3/3	PS 1	0	0	0	0	10	NA ^b^	NA
6	9/9	PS 1	1	0.1	1.11	0.111	10	NA	NA
7	9/9	PS 1	4	0.4	3.32	0.332	10	NA	NA
8	9/9	PS 1	8	0.8	6.55	0.655	10	NA	NA

^a^ Based on the analysis of initial samples only. ^b^ NA = Not Applicable

**Table 2:** Toxicokinetic parameters of PS **1** in rats.

Group	Pyroanalog 531 Dose (mg/kg)	Sex	T_max_ (h)	T_1/2_	Cmax (ng/mL)	AUC_last_ (h ng/mL)	AUC0_0_∞ (h * ng/mL)
6	1.11	M F	0.500 0.500	7.94 7.45	33,700 37,000	367,000 378,000	417,000 435,000
7	3.32	M F	0.083 0.083	7.03 9.13	111,000 128,000	1,180,000 1,340,000	1,290,000 1,600,000
8	6.55	M F	1.000 0.500	9.45 9.90	214,000 229,000	3,120,000 3,180,000	3,381,000 3,810,000

**Table 3:** Group assignments and dose levels (dog study).

	Number of Animals (M/F)	Test Article	Target		Actual ^a^		Dose Volume (mL/Kg)	Number of Animals for Necropsy (M/F)	
			Dose Level (mg/kg)	Dose Conc. (mg/mL)	Dose Level (mg/kg)	Dose Conc. (mg/kg)			
								Main (Day 2)	Recovery (Day 29)
Toxicity Groups									
1	6/6	PS **1**	0	0	0	0	5	3/3	3/3
2	6/6	PS **1**	0.5	0.1	0.42	0.0847	5	3/3	3/3
3	6/6	PS **1**	2	0.4	1.52	0.304	5	3/3	3/3
4	6/6	PS **1**	4	0.8	3.45	0.690	5	3/3	3/3
Toxicokinetic Groups									
5	6/6	PS **1**	0	0	0	0	5	NA	NA
6	6/6	PS **1**	0.5	0.1	0.42	0.0847	5	NA	NA
7	6/6	PS **1**	2	0.4	1.52	0.304	5	NA	NA
8	6/6	PS **1**	4	0.8	3.45	0.690	5	NA	NA

^a^ Based on the analysis of initial samples, only dose animals were administered 0 or 4 mg/kg of the test article in 2% Pluronic.

**Table 4:** Mean toxicokinetic parameters of PS **1** in dogs.

Group	Pyro Analog 531 Dose (mg/kg)	Sex	T_max_ (hr)	T_1/2_	Cmax (ng/ML)	AUC_last_ (hr*ng/mL
2	0.42	M F	0.458 0.153	NR 13.7	5330 7000	83,100 110,000
3	1.52	M F	0.389 0.083	NR NR	28,500 31,100	365,000 392,000
4	3.45	M F	1.153 0.3.75	NR NR	76,000 92,400	976,000 1,200,000

## Data Availability

The data presented in this manuscript are available on request to Pandey.
